# Circadian clocks and their role in kidney and eye diseases across organ systems

**DOI:** 10.3389/fphys.2025.1583502

**Published:** 2025-05-27

**Authors:** Xiuli Chen, Wenxiao Zhang, Yue Gu, Shenzhen Huang

**Affiliations:** ^1^ Department of Gynecology and Obstetrics, Henan Provincial People’s Hospital, People’s Hospital of Zhengzhou University, School of Clinical Medicine of Henan University, Zhengzhou, Henan, China; ^2^ Department of Ophthalmology, People’s Hospital of Zhengzhou University, Henan Provincial People’s Hospital, Zhengzhou, China; ^3^ Department of Nephrology, Henan Clinical Medical Research Center for Nephropathy, Henan Provincial Key Laboratory of Kidney Disease and Immunology, Henan Provincial People’s Hospital, Zhengzhou University People’s Hospital, Henan University People’s Hospital, Zhengzhou, Henan, China; ^4^ Department of Nephrology, Fuwai Central China Cardiovascular Hospital, Zhengzhou, Henan, China; ^5^ Henan Eye Institute, Henan Eye Hospital, People’s Hospital of Henan University, People’s Hospital of Zhengzhou University, Henan Provincial People’s Hospital, Zhengzhou, China

**Keywords:** circadian rhythms, kidney disease, eye disease, chronotherapy, inflammation

## Abstract

Circadian rhythms, the body’s intrinsic 24-h cycles, regulate essential physiological processes across multiple organ systems. Disruptions in these rhythms are increasingly recognized as significant contributors to chronic kidney disease and ocular diseases such as glaucoma, dry eye disease, and diabetic retinopathy. This review examines the interconnections between circadian rhythms in kidney and eye health, focusing on shared pathological pathways including oxidative stress, inflammation, and fibrosis. Current therapeutic strategies such as chronotherapy, light therapy, and time-restricted feeding show promise in mitigating disease progression by restoring circadian alignment. This review emphasizes integrated circadian-focused treatments to address these co-morbid conditions, paving the way for novel preventive and therapeutic interventions.

## Highlights

• Circadian rhythm disruptions contribute to kidney and eye disease pathogenesis.
• Emerging circadian-based therapies, including chronotherapy and light therapy, offer new therapeutic avenues.
• Integrated models are essential for assessing circadian disruption across organ systems.

## 1 Introduction

Circadian rhythms are endogenous 24-h cycles that regulate various physiological processes in humans, thereby ensuring optimal functioning across the day (Czeisler et al., 1999; Fagiani et al., 2022). These rhythms are regulated by the central clock located in the suprachiasmatic nucleus (SCN) of the hypothalamus, which synchronizes with environmental cues such as light and darkness, thus maintaining alignment with the 24-h day-night cycle (Patton and Hastings, 2018). At the molecular level, circadian rhythms are regulated by a network of clock genes, including *Circadian Locomotor Output Cycles Kaput (CLOCK), Brain and Muscle ARNT-Like 1 (BMAL1), Period (PER),* and *Cryptochrome (CRY)*, which orchestrate gene expression in a time-dependent manner and influence various biological functions. Disruption of these rhythms, caused by factors such as shift work, jet lag, or irregular sleep patterns, has been associated with a range of health issues, including metabolic disorders (Fagiani et al., 2022; Kalsbeek et al., 2011; Reinke and Asher, 2019), cardiovascular diseases (Chellappa et al., 2019a; Portaluppi et al., 2012a; Thosar et al., 2018), and neurodegenerative conditions (Colwell, 2021; Nassan and Videnovic, 2022; Leng et al., 2019).

In addition to central processes like sleep, circadian rhythms also extend to peripheral organs, including the liver (Tahara and Shibata, 2016; Weger et al., 2021), kidney (Firsov and Bonny, 2018a; Stow and Gumz, 2011a; Costello et al., 2022), and eye tissues (Wiechmann and Summers, 2008a; Huang et al., 2024a; Zou et al., 2023; Huang et al., 2022a; Huang et al., 2021), which contain their own circadian clocks. These peripheral clocks coordinate organ-specific functions, including glomerular filtration (Song et al., 2024), tubular reabsorption (Bignon et al., 2023), and electrolyte excretion (Costello et al., 2025) in the kidneys, as well as intraocular pressure (IOP) regulation (Ikegami et al., 2020), tear secretion (Huang et al., 2025), and photoreceptor maintenance in the eyes (Fagiani et al., 2022; Koronowski and Sassone-Corsi, 2021). Disruption of circadian rhythms in these organs can lead to significant health consequences, particularly in the context of chronic diseases (Reinke and Asher, 2019; Gale et al., 2011; Nakamura et al., 2011). There is increasing recognition that aligning circadian biology with physiological rhythms may enhance treatment efficacy for both renal and ocular diseases (Fagiani et al., 2022). Research into chronotherapy and other clock-based interventions reflects this emerging interest in circadian-targeted therapies (Masri and Sassone-Corsi, 2013; Volkmann et al., 2024; Albrecht, 2012; Hou et al., 2020). Importantly, the kidney and eye, though functionally distinct, exhibit striking similarities in their circadian regulation of physiological processes and vulnerability to clock disruption. Both organs rely on tightly controlled diurnal rhythms to maintain homeostasis—such as glomerular filtration and electrolyte reabsorption in the kidney, and IOP and photoreceptor renewal in the eye (Song et al., 2024; Bignon et al., 2023; Ikegami et al., 2020; Ohashi et al., 2017). Circadian misalignment can thus trigger overlapping pathological cascades in both systems.

The kidney serves as a model organ for studying circadian regulation of filtration, electrolyte transport, and hormonal signaling, and circadian disturbances are increasingly recognized as contributors to renal pathology (Ohashi et al., 2017; Olaoye et al., 2019). Chronic kidney disease (CKD) constitutes a global health crisis, with estimates indicating that approximately 10%–16% of adults worldwide are affected by some form of kidney dysfunction (Coresh et al., 2007; Kazancioğlu, 2013; Francis et al., 2024). CKD is characterized by a gradual decline in renal function, which can progress to end-stage renal disease, ultimately requiring dialysis or kidney transplantation. The increasing prevalence of CKD is linked to risk factors, including diabetes, hypertension, obesity, and an aging population (Sundström et al., 2022; Couser et al., 2011). The burden of CKD extends beyond the physical deterioration of renal function, encompassing broader psychosocial impacts on patients, which significantly impair quality of life. Individuals with kidney failure often experience physical debilitation, cognitive dysfunction, and a reduced ability to perform daily tasks, also imposing substantial economic costs (Bello et al., 2015; Pippias et al., 2024; Vivante, 2024).

Similarly, ocular tissues such as the retina and cornea harbor robust circadian clocks that regulate visual processing, tear secretion, and retinal metabolism—processes that are also susceptible to circadian misalignment. Ophthalmological disorders, including age-related macular degeneration (AMD), diabetic retinopathy (DR), and glaucoma, are major causes of blindness and visual impairment worldwide (Wong WL. et al., 2014). As the population ages and the incidence of chronic diseases such as diabetes increases, the prevalence of these diseases is expected to rise. The impact on patients' lives is substantial, as vision loss results in reduced social interaction, independence, and mental wellbeing. Emerging evidence suggests that circadian rhythm disruption contributes to the pathogenesis of both kidney and eye diseases, as disruptions to biological clocks exacerbate inflammation, oxidative stress, and metabolic dysfunction in these organs (Firsov and Bonny, 2018b; McMahon et al., 2014; Bhoi et al., 2023). Understanding the interrelationship between circadian rhythms and these disorders could, therefore, lead to novel approaches for prevention and treatment.

Given the temporal regulation of physiological processes in both kidney and eye tissues, there is increasing interest in targeting circadian clocks for therapeutic benefit. Chronotherapeutic strategies—such as timed drug delivery, light therapy, and feeding schedules—are being actively explored to enhance treatment efficacy and minimize side effects in kidney and eye diseases (De Lavallaz and Musso, 2018; Thomas and Cooper, 2010; Zhu et al., 2021; Chaix et al., 2014).

Mounting evidence implicates circadian rhythm disruption in the pathogenesis of both CKD and ocular disorders such as glaucoma, DR, and dry eye disease (DED) (Paul et al., 2009; Liu WJ. et al., 2023; Wong CW. et al., 2014). Misalignment between central and peripheral clocks exacerbates inflammation, oxidative stress, and metabolic dysfunction in renal and ocular tissues. This review examines how circadian dysregulation contributes to disease onset and progression, with a particular focus on shared pathological pathways and emerging circadian-based therapeutic strategies. Furthermore, the review evaluates emerging therapeutic strategies aimed at restoring circadian synchronization in patients with kidney and eye diseases. These include approaches such as light therapy, pharmacological agents, and behavioral modifications that have shown potential in mitigating the harmful effects of circadian misalignment (Dodson and Zee, 2010; Faulkner et al., 2020; Lee et al., 2021; Ruan et al., 2021). Despite promising results, further research is needed to identify effective, evidence-based interventions for clinical application. Finally, the review highlights gaps in current research, particularly regarding the molecular mechanisms underlying circadian regulation in these organs, and proposes future directions for investigation.

In summary, this review provides a comprehensive synthesis of circadian biology in kidney and eye health, aiming to identify novel therapeutic targets and strategies. By elucidating the connections between circadian rhythms and these disorders, this review aims to pave the way for improved preventive and therapeutic interventions, ultimately enhancing patient outcomes and quality of life.

## 2 Physiological mechanisms of circadian rhythms

### 2.1 Molecular regulation of circadian rhythms

The regulation of circadian rhythms is a dynamic process controlled by a complex biological clock, which synchronizes various physiological functions with the 24-h day-night cycle. The SCN, located in the hypothalamus, functions as the body’s “master clock” (Patton and Hastings, 2018). This central regulator receives direct light input via the retinohypothalamic tract (RHT), enabling alignment of the internal clock with environmental light-dark cues (Moore, 2013). Synchronization is crucial for optimizing physiological functions, ensuring that processes occur at the most appropriate times of day, which is essential for maintaining homeostasis.

**FIGURE 1:**
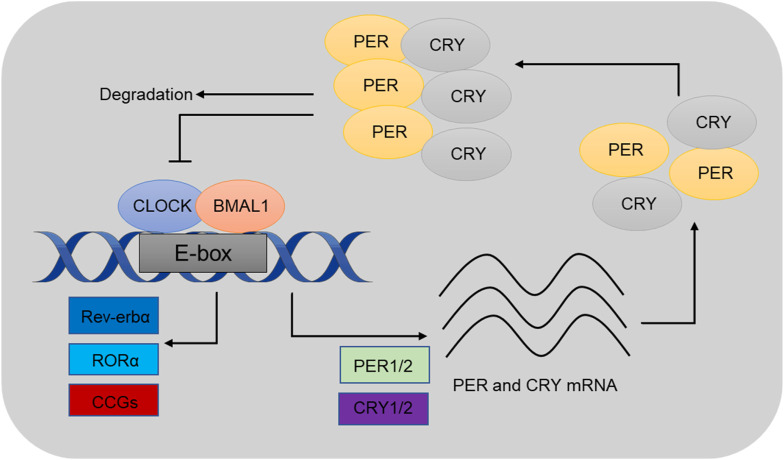
Schematics illustrating a 24-h cell-autonomous circadian clock cycle. At circadian dawn, Clock and Bmal1 (positive regulators) dimerize to activate transcription of Per and Cry (negative regulators) through the enhancer box regulatory sequence. These genes are known as clock-controlled genes (CCGs), and their expression regulates various physiological processes. At the end of the circadian day, Per and Cry proteins move into the nucleus to inhibit their own expression. As circadian night progresses, the dimer is degraded, allowing the cycle to restart at dawn. Reproduced from huang (Huang et al., 2020) and Bhatwadekar (Bhatwadekar and Rameswara, 2020).

At the molecular level, circadian rhythms are governed by transcription-translation feedback loops (TTFLs), in which core clock genes, such as *Clock*, *Bmal1*, *Per1*, *Per2*, *Cry1*, and *Cry2*, play key roles (Hurley et al., 2016; Tomita et al., 2005). The proteins encoded by these genes establish a feedback system that generates oscillations. CLOCK and BMAL1 form a dimer that activates the transcription of *Per* and *Cry* genes, whose protein products (PER and CRY) subsequently inhibit CLOCK-BMAL1 activity, thereby completing the feedback cycle (Patke et al., 2020; Partch et al., 2014) (Figure 1). These oscillations regulate a broad range of physiological processes, including sleep-wake cycles, metabolism, and cellular repair.

### 2.2 Role of circadian rhythms in overall health

Circadian rhythms are crucial for regulating various physiological processes, and disruptions in these rhythms can significantly affect overall health, particularly in terms of metabolism (Johnston et al., 2016), immune function (Scheiermann et al., 2013), and cardiovascular health (Portaluppi et al., 2012b). Furthermore, recent research has increasingly emphasized the role of circadian rhythms in the development and progression of diseases in specific organs, such as the kidney (Firsov and Bonny, 2018b) and eyes (Wiechmann and Summers, 2008b).

In the kidney, circadian rhythms regulate essential processes, including sodium balance, glomerular filtration, and renal blood flow (Firsov and Bonny, 2018a; Firsov and Bonny, 2018b). Kidney cells harbor intrinsic circadian clocks that regulate renal function at the molecular level. Disruptions in these rhythms, as observed in conditions such as shift work or chronic jet lag, are associated with altered kidney function, including impaired sodium homeostasis, elevated blood pressure, and a potential progression to CKD (Costello et al., 2022; Juffre and Gumz, 2024). Studies have shown that clock gene expression is dysregulated in kidney tissues under pathological conditions, contributing to disease progression. For instance, Bmal1 knockout mice exhibit renal damage resulting from altered circadian regulation of cellular repair mechanisms (Zhang and Pollock, 2018). Moreover, circadian disruption has been associated with an increased susceptibility to acute kidney injury (AKI), underscoring the importance of rhythmicity in maintaining renal health (Hill et al., 2021; Motohashi et al., 2020).

In the eye, circadian rhythms are essential for regulating the functions of the retina and lacrimal glands, which are responsible for vision and tear production, respectively. The retinal clock governs light-dark adaptation and regulates the expression of genes involved in photoreception and retinal cellular repair (McMahon et al., 2014; Paul et al., 2009). Disruptions in circadian rhythms can lead to DED and retinal degeneration, conditions increasingly recognized in shift workers and individuals with irregular sleep patterns (Parravano et al., 2022; Jauregui-Lozano et al., 2022). Melatonin, which peaks during the night, plays a role in regulating eye health by protecting the retina from oxidative stress and modulating retinal cell functions (Felder-Schmittbuhl et al., 2024). Moreover, circadian misalignment is associated with an increased risk of conditions such as glaucoma and DR (Bhatwadekar and Rameswara, 2020; Jean-Louis et al., 2008).

Beyond the kidney and eye, circadian rhythms exert systemic control over key physiological domains. In the metabolic system, the circadian clock regulates hepatic gluconeogenesis, insulin sensitivity, and adipocyte function; circadian disruption is associated with metabolic syndrome, obesity, and type 2 diabetes (Froy, 2010). In the cardiovascular system, daily oscillations in blood pressure and heart rate are governed by circadian rhythms, and their disruption elevates the risk of hypertension and myocardial infarction (Portaluppi et al., 2012b). The immune system is also rhythmically controlled, with time-of-day–dependent variations in leukocyte trafficking, cytokine production, and immune surveillance; loss of circadian control can impair host defense and contribute to chronic inflammatory diseases (Wang et al., 2022). In the central nervous system, circadian misalignment affects sleep-wake cycles, neuroplasticity, and cognitive performance, and has been implicated in the pathogenesis of mood disorders and neurodegeneration (Musiek and Holtzman, 2016).

Circadian rhythms synchronize essential processes, including metabolism, immune response, and organ function. The role of circadian rhythms in disease pathogenesis is becoming increasingly evident in the kidney and eye. Disruption of these rhythms can exacerbate various health conditions, including metabolic disorders, kidney dysfunction, and eye diseases. Therefore, the circadian timing system is crucial for maintaining health and preventing disease.

## 3 Circadian rhythms in kidney function

### 3.1 Circadian regulation of kidney physiology

**FIGURE 2:**
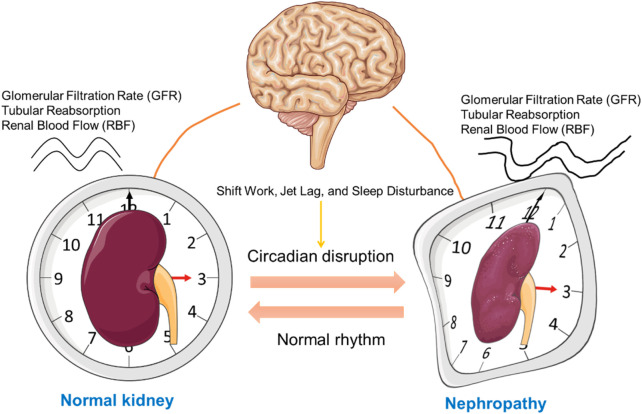
Influence of Circadian Rhythms on Kidney Function and Nephropathy Progression. This schematic illustrates the relationship between circadian rhythm regulation by the central nervous system (represented by the brain) and kidney function. On the left, a normal kidney is displayed within a clock face, symbolizing regular circadian control over renal function. In contrast, the right side shows a kidney affected by nephropathy, with a distorted clock face, indicating disrupted circadian alignment. The bidirectional arrows between the brain and kidneys represent the mutual influence between the central circadian pacemaker and kidney health, emphasizing the impact of circadian misalignment on the progression of kidney disease. This figure was created using the Servier Medical ART: SMART (smart.servier.com) according to a Creative Commons Attribution 3.0.

The kidney, a vital organ responsible for regulating fluid balance, waste elimination, and electrolyte homeostasis, operates under the governance of circadian rhythms. Several renal functions, including glomerular filtration rate (GFR), tubular reabsorption, and renal blood flow (RBF), exhibit circadian variation (Firsov and Bonny, 2018a; Firsov and Bonny, 2018b) (Figure 2). GFR, a crucial marker of kidney function, peaks during the active phase of the diurnal cycle—typically during the day in humans—and declines at night (Ansermet et al., 2019; Levey et al., 2015). This daily rhythm is thought to align with the body’s metabolic demands and hydration status. Similarly, the tubular reabsorption of water, sodium, potassium, and other solutes also follows a circadian pattern, optimizing renal handling of electrolytes and waste products according to the body’s circadian needs (Firsov and Bonny, 2018b).

At the molecular level, the circadian regulation of kidney physiology is intricately governed by a network of clock genes, including *BMAL1*, *CLOCK*, *PER*, and *CRY*. These genes form the core of the intrinsic molecular machinery that coordinates cellular processes in renal tissues. Specifically, BMAL1 and CLOCK, as positive regulators, initiate the transcription of target genes involved in various renal functions, such as sodium reabsorption, vascular tone, and blood pressure regulation, all of which are influenced by circadian rhythms. BMAL1 and CLOCK regulate a host of clock-controlled genes (CCGs) involved in renal metabolism, inflammation, and electrolyte handling (Costello et al., 2022). These include genes encoding for sodium/hydrogen exchangers (e.g., NHE3), epithelial sodium channels (ENaC), aquaporins (AQP2), and renin-angiotensin system components. Through rhythmic transcriptional regulation, circadian clocks synchronize these effector pathways with behavioral and metabolic cycles, ensuring optimal kidney performance. Conversely, PER and CRY proteins establish a negative feedback loop to regulate the amplitude and phase of the clock gene oscillations, ensuring that renal processes are precisely timed (Costello et al., 2022; Johnston and Pollock, 2018).

Emerging evidence demonstrates that circadian clocks exert cell type-specific regulatory control within the kidney. In podocytes, BMAL1 is crucial for maintaining structural integrity and cellular homeostasis. Podocyte-specific deletion of BMAL1 leads to glomerular injury, foot process effacement, and albuminuria, highlighting its role in preserving glomerular filtration barrier function (Wang et al., 2024). Similarly, circadian regulation in proximal tubular cells influences sodium transporter expression and mitochondrial metabolism, while collecting duct principal cells rely on clock genes to control water reabsorption through vasopressin signaling pathways (Pearce et al., 2015). These findings reveal that renal clocks operate in a compartmentalized manner, orchestrating time-of-day–dependent gene expression to support nephron function and homeostasis.

### 3.2 Circadian rhythms in renal pathophysiology

Circadian rhythms are not only essential for maintaining normal kidney function but also play a significant role in the progression of kidney diseases. The impact of circadian rhythms on conditions such as CKD, diabetic nephropathy, and hypertension-related kidney injury has been well-documented in both experimental models and clinical studies (Ohashi et al., 2017; Olaoye et al., 2019; Ix et al., 2014; Hsu et al., 2012; Hansen et al., 1996; Isobe et al., 2015). Circadian variation in kidney disease progression is evident through fluctuations in biomarkers, such as serum creatinine and urinary albumin excretion, which exhibit peak values at specific times of the day (Nishijima et al., 2014; Koopman et al., 1989). This suggests that kidney damage, particularly in CKD, may be more pronounced during certain phases of the circadian cycle, potentially aligning with metabolic stressors or fluctuations in blood pressure.

Research indicates that diabetic nephropathy follows a circadian pattern in glomerular filtration and tubular reabsorption, with peak renal dysfunction correlating with periods of heightened insulin resistance or elevated blood glucose levels (Olaoye et al., 2019; Johnston and Pollock, 2018). For instance, in animal models, nocturnal hyperglycemia often exacerbates renal injury, while daytime variations in insulin sensitivity are associated with impaired renal function (Noshahr et al., 2020; Wu et al., 2023). Similarly, studies on hypertensive nephropathy have demonstrated that the kidney’s circadian rhythm influences blood pressure regulation and that renal tissues are more vulnerable to damage during phases of circadian misalignment (Firsov and Bonny, 2018b; Costello and Gumz, 2021).

Additionally, circadian rhythms play a crucial role in the development of kidney fibrosis, a hallmark feature of CKD and other renal pathologies (Stow and Gumz, 2011a; Liu C. et al., 2023). Fibrosis is primarily driven by transforming growth factor-beta (TGF-β), a key regulator of extracellular matrix production and fibrosis progression. Research has shown that TGF-β expression oscillates in a time-dependent manner in renal tissues, with peaks aligning with the kidney’s active repair and regeneration phases (Chen et al., 2015; Han et al., 2020). The circadian regulation of collagen I/III expression in kidney tubules and interstitial spaces further underscores the rhythmic nature of kidney fibrosis (Chen et al., 2015; Preston et al., 2022). Inhibition of the circadian rhythm in kidney fibroblasts has been shown to accelerate fibrosis progression (Desvergne et al., 2016; Egstrand et al., 2020), highlighting the critical importance of maintaining normal circadian signaling to preserve kidney health.

### 3.3 Circadian rhythm disruptions and kidney diseases

Circadian misalignment, induced by factors such as shift work, jet lag, and sleep disturbances, has emerged as a significant risk factor for kidney disease progression (Kim et al., 2024; Cho et al., 2023; Ricardo et al., 2017). Chronic disruptions to the sleep-wake cycle, as seen in shift workers, are associated with increased prevalence of hypertension and CDK (Turek et al., 2012). These effects are largely mediated by altered renal hemodynamics and dysregulation of hormonal pathways such as the renin-angiotensin-aldosterone system (RAAS) (Ricardo et al., 2017; Uhm et al., 2018). Specifically, misalignment of circadian rhythms in shift workers is linked to elevated blood pressure and worsened kidney outcomes, likely through dysregulation of the RAAS, a key regulator of renal function.

Animal models have provided valuable insights into the impact of circadian disruption on kidney function (Koch et al., 2009). In rodent models subjected to chronic circadian disruption, such as light-dark cycle inversion, researchers have observed impaired renal function, increased albuminuria, and exacerbated kidney fibrosis (Zhang et al., 2024; Mohandas et al., 2022). These findings have been corroborated by human cohort studies, which show that individuals with irregular sleep patterns are at a higher risk of kidney disease, including hypertensive nephropathy and diabetic kidney disease. At the molecular level, circadian disruption affects the regulation of core clock genes, leading to aberrant gene expression in renal tissues. Disrupted expression or mutations in core clock genes such as *BMAL1*, *PER*, and *CRY* results in altered expression of genes involved in inflammation, fibrosis, and oxidative stress (Abe et al., 2022; Yang et al., 2024). For example, circadian misalignment dysregulates the TGF-β signaling pathway, promoting excessive collagen deposition and fibrosis in renal tissues (Raza et al., 2022; Xing et al., 2022). Similarly, disruption of circadian rhythms increases the production of reactive oxygen species (ROS), which accelerates tubular injury, glomerulosclerosis, and fibrosis (Costello et al., 2022; Zhang et al., 2024; Esposito et al., 2020; Hervé et al., 2024).

Beyond hypertensive and diabetic kidney disease, circadian disruption has also been implicated in other renal conditions. For instance, in animal models of glomerulonephritis, disrupted expression of clock genes such as BMAL1 and PER1 correlates with heightened inflammatory responses and glomerular injury (Costello et al., 2022). Furthermore, chronobiological factors influence urinary pH, calcium excretion, and oxalate levels—key determinants of kidney stone formation. Night-shift workers and individuals with irregular sleep patterns may be at increased risk of nephrolithiasis due to dysregulated mineral metabolism and changes in urine supersaturation (He et al., 2023). These findings indicate that circadian dysregulation broadly affects renal health across a spectrum of kidney diseases.

Beyond molecular and hormonal changes, circadian disruption also impacts the sympathetic nervous system (SNS), which plays a crucial role in regulating renal hemodynamics (Logan et al., 2011). Dysregulation of the SNS, induced by altered sleep patterns and circadian misalignment, can result in increased blood pressure and impaired renal blood flow, further contributing to kidney damage. Studies have shown that circadian misalignment leads to endothelial dysfunction, exacerbating renal injury and accelerating disease progression (Hill et al., 2021; Crnko et al., 2018).

In conclusion, the relationship between circadian rhythms and kidney function is complex and multifaceted. Disruptions in the kidney’s intrinsic circadian regulation led to a cascade of pathophysiological processes, including fibrosis, inflammation, and oxidative stress, all of which contribute to kidney disease progression. Furthermore, the detrimental effects of circadian disruption on kidney function are amplified in individuals with pre-existing conditions, such as hypertension or diabetes. Understanding the intricate molecular mechanisms behind circadian regulation in kidney physiology and pathology offers valuable insights into potential therapeutic interventions aimed at improving renal health.

## 4 Circadian rhythms in eye health and disease

### 4.1 Circadian regulation of ocular tissue functions

**FIGURE 3:**
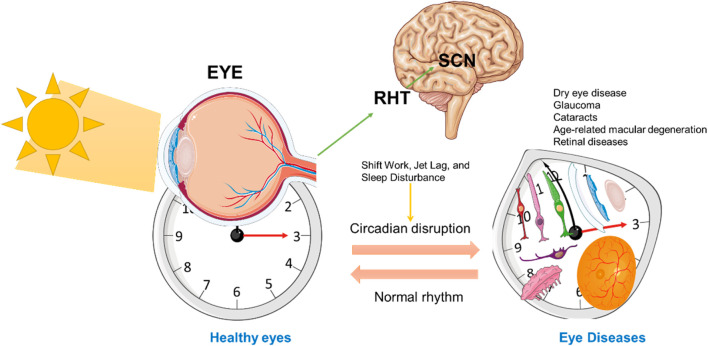
Circadian Regulation of Ocular Function and Retinal Health. This schematic represents the influence of circadian rhythms on ocular function, particularly in the retina, through signals from the central suprachiasmatic nucleus (SCN) in the brain. The left side illustrates a normal eye exposed to sunlight, with signals transmitted to the SCN to synchronize the circadian clock. The RHT is directly involved in the transfer of light-based input from the retina to the SCN. On the right, an altered or misaligned circadian rhythm is depicted, showing various ocular tissue (including photoreceptors, bipolar cells, ganglion cells, RPE, cornea, retina, and lacrimal gland) under a distorted clock face. The bidirectional arrows between the SCN and eye represent the mutual feedback loop of circadian regulation affecting cellular processes critical for maintaining eye health. This figure was created using the Servier Medical ART: SMART (smart.servier.com) according to a Creative Commons Attribution 3.0.

Circadian rhythms are essential in regulating various functions across specific ocular tissues, including the ciliary body, lacrimal glands, and retina, each following distinct diurnal patterns (Wiechmann and Summers, 2008b; Felder-Schmittbuhl et al., 2018; Zhang and Jie, 2024). These tissue-specific rhythms align with the body’s internal biological clock, optimizing visual performance during waking hours and facilitating recovery during sleep (Figure 3).

Among these regulated processes, IOP is one of the most extensively studied circadian phenomena in ocular health. IOP follows a diurnal fluctuation, peaking in the early morning and gradually decreasing throughout the day (Krishna et al., 1995; Del Sole et al., 2007; Ikegami, 2024). This variation is largely managed by circadian regulation within the ciliary body and trabecular meshwork, where clock genes influence the production and outflow of aqueous humor. Disruptions to this circadian rhythm, which can lead to persistently elevated IOP, are associated with glaucoma, a condition that poses risks of optic nerve damage and visual impairment (Gubin et al., 2021; Ciulla et al., 2020).

Similarly, tear production by the lacrimal glands follows a circadian rhythm, with secretion rates increasing during waking hours to maintain ocular surface hydration, and decreasing during sleep (Huang et al., 2022a; Vu et al., 2021; Webber et al., 1987). This rhythm is controlled by hormonal signals, such as melatonin and vasoactive intestinal peptide (VIP). Circadian disruptions, often due to night shift work or irregular sleep, are associated with DED, a condition marked by reduced tear production and ocular surface irritation (Li et al., 2018; Zeng et al., 2024).

The retina, another key tissue in ocular function, is also regulated by circadian rhythms. Retinal function peaks during daylight hours, supporting optimal photoreceptor activity, gene expression, and blood flow, and decreases at night. Clock genes such as *BMAL1*, *CLOCK*, *PER*, and *CRY* synchronize these cellular processes with the light-dark cycle (Huang et al., 2024a; Bhoi et al., 2023; DeVera and Tosini, 2020; Ye et al., 2023). Disturbances in these rhythms are implicated in retinal diseases, including AMD and DR (Liu WJ. et al., 2023; Fanjul-Moles and López-Riquelme, 2016).

Circadian rhythms also govern other tissue-specific functions, such as protein synthesis and degradation in the lens, which are crucial for maintaining lens transparency. Disruptions in these processes are linked to cataract formation, characterized by lens clouding and vision impairment (Yan and Wang, 2016; Chellappa et al., 2019b).

### 4.2 Circadian rhythm disruptions and common eye diseases

Circadian rhythm disruptions are closely associated with the development and progression of several ocular diseases, including DED (Huang et al., 2021; Zeng et al., 2024), glaucoma (Faulkner et al., 2020), cataracts (Turner et al., 2010), and retinal diseases (McMahon et al., 2014). Misalignment between the internal circadian clock and environmental cues, such as that caused by shift work, jet lag, and chronic sleep disorders, alters crucial ocular functions—particularly IOP, tear production, and retinal function—exacerbating disease pathogenesis.

Animal studies have highlighted the role of circadian rhythms in lacrimal gland function, showing that mice exposed to continuous light or irregular light-dark cycles exhibit reduced tear secretion, increased ocular surface inflammation, and cytokine imbalances, resembling DED symptoms (Li et al., 2018; Zeng et al., 2024). Our previous results showed that the light cycle phase advance as a model for jet lag and sleep loss led to lacrimal gland function impairment and disturbance of tear secretion rhythm in mice (Huang et al., 2022a; Huang et al., 2021). Clinical studies similarly report higher incidences of DED among individuals with irregular sleep patterns or artificial light exposure, underscoring the importance of circadian regulation in immune responses on the ocular surface (Rolando et al., 2024; Kawashima et al., 2016).

Circadian rhythms are also implicated in glaucoma, primarily through their influence on diurnal IOP fluctuations. Studies in animal models, such as rabbits exposed to constant light, demonstrate elevated IOP, a factor known to affect optic nerve health adversely (Rowland et al., 1981). Clinically, individuals with disrupted sleep-wake cycles, including shift workers, often experience sustained IOP elevations, which are correlated with an increased risk of glaucoma progression and optic nerve damage (Kara and Yilmaz, 2015; Wang et al., 2013).

Retinal diseases like AMD and DR are further affected by circadian disruptions. In AMD models, the circadian regulation of oxidative stress and protein turnover in retinal pigment epithelium (RPE) cells has been shown to mitigate cellular damage (Fanjul-Moles and López-Riquelme, 2016; Baba et al., 2022a). When these rhythms are disrupted, as evidenced in both animal and clinical studies, oxidative stress, vascular dysfunction, and retinal inflammation increase, accelerating disease progression (Schmoll et al., 2011).

Retinitis pigmentosa (RP), a group of inherited retinal dystrophies, has also been linked to circadian disturbances. RP affects photoreceptor cells, which are critical not only for vision but also for conveying light signals to the SCN, the central circadian pacemaker. Studies have shown that patients with RP often exhibit sleep disturbances and altered melatonin rhythms, suggesting a bidirectional relationship between photoreceptor degeneration and circadian misalignment (Agorastos and Huber, 2011). Moreover, animal models of RP have revealed impaired light-mediated entrainment of circadian rhythms and disrupted expression of retinal clock genes such as PER1 and CRY1, highlighting the integral role of retinal integrity in maintaining systemic circadian homeostasis (Guido et al., 2010). These findings underscore the importance of circadian regulation in the management of RP and suggest potential therapeutic benefits of circadian-based interventions.

Research on circadian rhythms in cataract formation also reveals that lens transparency is maintained by rhythmic protein synthesis. Animal studies indicate that circadian misalignment leads to protein aggregation in the lens, initiating cataractogenesis (Yan and Wang, 2016). In human studies, older individuals with a history of circadian disruption are more likely to develop cataracts, suggesting a cumulative effect of misalignment on lens opacity over time (Yan and Wang, 2016; Saeki et al., 2014).

In summary, substantial evidence from animal models and clinical research demonstrates that circadian rhythms are essential for ocular health. Disruptions in these rhythms are implicated in various eye diseases, highlighting the potential of circadian-targeted therapies to improve outcomes in conditions such as DED, glaucoma, retinal diseases, and cataracts.

## 5 Interlinking circadian rhythms in kidney and eye diseases

**FIGURE 4:**
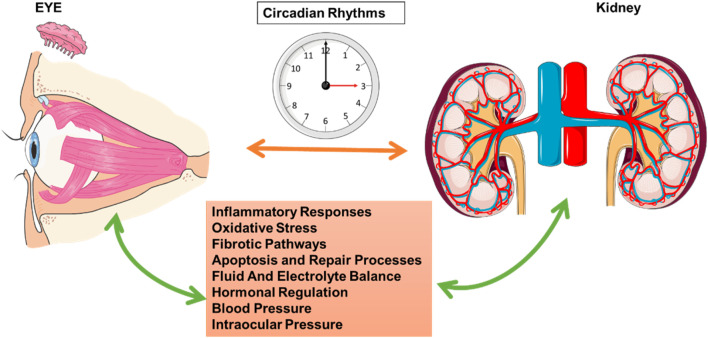
Circadian Rhythms and Interactions between Ocular and Renal Functions. This schematic highlights the role of circadian rhythms in regulating physiological processes that link ocular and renal functions. The central clock image represents the circadian rhythm, which coordinates multiple biological functions across different organs. The eye on the left and the kidney on the right are interconnected through circadian mechanisms that influence inflammatory responses, oxidative stress, fibrotic pathways, apoptosis and repair processes, fluid and electrolyte balance, hormonal regulation, blood pressure, and IOP. Bidirectional arrows illustrate the mutual influence and synchronization of these processes, emphasizing the interdependence of circadian-regulated pathways in maintaining both ocular and renal health. This figure was created using the Servier Medical ART: SMART (smart.servier.com) according to a Creative Commons Attribution 3.0.

Kidney and eye health are intricately regulated by circadian rhythms, and disruptions in these rhythms impact several shared mechanisms, thereby increasing disease susceptibility in both organs. Research has shown that circadian regulation is crucial for inflammatory responses, oxidative stress control, fibrotic pathways, apoptosis and repair processes, fluid and electrolyte balance, hormonal regulation, and pressure regulation, including blood pressure and IOP (Figure 4). Understanding these interconnected mechanisms provides insight into the broader implications of circadian misalignment for kidney and eye pathophysiology.

### 5.1 Shared mechanisms between kidney and eye pathophysiology

#### 5.1.1 Inflammatory pathways in circadian dysfunction

Inflammation is a central driver of tissue injury in both the kidney and eye (Wong CW. et al., 2014). Circadian clocks modulate inflammatory tone by controlling the rhythmic expression of immune-related genes. Core clock proteins such as BMAL1 and CLOCK regulate nuclear factor kappa B (NF-κB) signaling and downstream proinflammatory cytokines like TNF-α, IL-6, and IL-1β. Loss of BMAL1 enhances nuclear factor kappa-light-chain-enhancer of activated b cells (NF-κB) activation and prolongs inflammatory gene transcription in both macrophages and renal epithelial cells (Curtis et al., 2014; Early et al., 2018).

In the kidney, circadian misalignment—induced by altered light-dark cycles or genetic manipulation—leads to increased macrophage infiltration, tubular injury, and glomerular inflammation. BMAL1-deficient mice show elevated expression of IL-6 and chemokines (e.g., CCL2), contributing to chronic kidney injury (Crislip et al., 2023; Nechemia-Arbely et al., 2008). In the eye, circadian disruption enhances retinal microglial activation and elevates inflammatory cytokines, accelerating progression of DR and AMD. CLOCK deficiency in retinal pigment epithelial cells has been shown to impair anti-inflammatory responses and compromise retinal barrier integrity (Baba et al., 2022b; George et al., 2021).

Collectively, these findings suggest that the circadian clock acts as an anti-inflammatory rheostat, and its misalignment can amplify immune-mediated injury in both the kidney and the eye.

#### 5.1.2 Oxidative stress pathways in circadian dysfunction

Circadian rhythms tightly regulate cellular redox homeostasis. Key antioxidant enzymes, including superoxide dismutase (SOD), catalase, and glutathione peroxidase, show rhythmic expression driven by BMAL1 and CLOCK. Disruption of circadian rhythms decreases the transcription of these enzymes, reducing the cell’s capacity to neutralize ROS (Mezhnina et al., 2022; Budkowska et al., 2022).

In the kidney, *Per1*-or *Bmal1*-deficient mice exhibit increased ROS accumulation and mitochondrial dysfunction, accelerating kidney injury (Crislip et al., 2018; Stow and Gumz, 2011b). Many renal transport genes have been identified as clock-controlled genes and have been linked to mitochondrial uncoupling and ROS production in renal cells (Firsov and Bonny, 2018b; Stow and Gumz, 2011b). In ocular tissues, the retina is particularly susceptible to oxidative damage due to high oxygen consumption and light exposure. Circadian misalignment impairs the rhythmic expression of NRF2, a master regulator of antioxidant defense, leading to increased oxidative stress and photoreceptor cell death in models of light-induced retinal degeneration (Desvergne et al., 2016; Salceda, 2024; Crooke et al., 2017; Lim et al., 2022).

These findings highlight a conserved role for circadian clocks in modulating antioxidant pathways, with implications for chronic disease progression in both kidney and eye.

#### 5.1.3 Circadian rhythms and fibrosis

Pathological fibrosis, characterized by abnormal extracellular matrix accumulation, is a common consequence in the progression of both kidney and eye diseases. Fibrosis is central to CKD (Huang et al., 2023), while in the eye, fibrotic changes contribute to conditions such as glaucoma and macular fibrosis (Friedlander, 2007). Circadian genes play a role in regulating fibrotic cytokines, including TGF-β and connective tissue growth factor (CTGF), which influence collagen deposition and the remodeling of the extracellular matrix. In the kidney, circadian CLOCK protein has been shown to directly facilitate fibrotic signaling through the activation of cyclooxygenase-2 (COX2), which in turn promotes TGF-β pathway activation. Notably, CLOCK enhances COX2 transcriptional activity in renal tubular epithelial cells, thereby elevating prostaglandin E2 levels and stimulating downstream TGF-β signaling (Chen et al., 2015). This molecular axis contributes to the initiation and propagation of renal fibrosis, particularly under conditions of circadian rhythm disruption. Circadian disruption impairs these regulatory processes, potentially leading to excessive fibrosis in both organs and contributing to functional decline (Chen et al., 2015; Dong et al., 2016).

Connective tissue growth factor (CTGF, also known as CCN2) is a critical downstream effector of TGF-β signaling and is significantly overexpressed in fibrotic regions of diseased kidneys. CTGF amplifies fibrotic responses by stimulating fibroblast proliferation, myofibroblast differentiation, and extracellular matrix (ECM) deposition. In experimental models of kidney disease, elevated CTGF levels are consistently observed in glomeruli and interstitial compartments, closely correlating with the severity of fibrosis and decline in renal function (Toda et al., 2018). Circadian misalignment may further potentiate CTGF overexpression, compounding fibrotic injury through sustained activation of profibrotic gene programs.

#### 5.1.4 Impact of circadian rhythm on cell apoptosis and repair mechanisms

The regulation of apoptosis and cellular repair mechanisms is also circadian-dependent in both kidney and retinal tissues. In the kidney, circadian cues regulate cell renewal processes essential for nephron maintenance (Firsov and Bonny, 2010), while in the retina, photoreceptor turnover is guided by circadian timing (Bhoi et al., 2023). Disruption of these rhythms accelerates cellular aging and diminishes repair capacity, thereby heightening susceptibility to diseases such as CKD and AMD (Firsov and Bonny, 2018b; Vallée et al., 2020).

#### 5.1.5 Circadian influence on fluid and electrolyte balance

The kidney and eye both rely on circadian rhythms to regulate fluid and electrolyte homeostasis. Circadian rhythms influence renal electrolyte excretion, which affects blood osmolarity and indirectly influences ocular hydration and IOP regulation (Mills, 1966; Martínez-Águila et al., 2021a). Disruptions in circadian regulation of fluid balance may adversely affect kidney function and contribute to ocular conditions such as glaucoma and DED.

#### 5.1.6 Hormonal regulation and diurnal rhythms

Hormonal signals, including melatonin and cortisol, are integral to circadian regulation and exert significant influence on kidney and eye physiology. Melatonin (Arendt, 2000), synthesized by the pineal gland in response to darkness, not only facilitates sleep initiation and maintenance but also serves as a potent antioxidant, protecting retinal and renal tissues from oxidative damage (Wiechmann and Summers, 2008a; Felder-Schmittbuhl et al., 2024; Han et al., 2020). Its secretion is light-sensitive and often diminished by circadian disruption, contributing to increased risks of kidney injury and retinal degeneration. Cortisol, peaking in the early morning and tapering throughout the day, modulates stress responses and impacts renal and ocular function (Sharma et al., 1989; James et al., 2007). Disruptions in cortisol rhythmicity have been associated with increased susceptibility to stress-induced damage in both systems. Together, these hormones exemplify how the circadian system orchestrates endocrine rhythms to maintain organ health and homeostasis (James et al., 2007).

#### 5.1.7 Shared circadian regulation of blood pressure and IOP

Both blood pressure and IOP are regulated by circadian rhythms, with dysregulation in these systems potentially leading to hypertension and glaucoma, respectively. The kidney regulates blood pressure via circadian-driven sodium reabsorption and the RAAS (Ohashi et al., 2017), while circadian rhythms control IOP through variations in aqueous humor dynamics within the eye (Martínez-Águila et al., 2021b). Misalignment of these rhythms disrupts pressure regulation, fostering conditions conducive to hypertension in the kidney and elevated IOP in the eye, thereby heightening the risk of related diseases.

Synthesizing evidence on these interconnected mechanisms reveals that circadian rhythms are crucial for maintaining health and preventing disease in both the kidney and the eye. Since circadian misalignment exacerbates inflammation, oxidative stress, fibrosis, and pressure dysregulation, circadian-based approaches should be considered in disease management and therapeutic interventions to improve kidney and eye health concurrently.

### 5.2 Circadian rhythms and disease co-morbidities

The relationship between kidney and eye diseases is especially notable in individuals with diabetes, where diabetic nephropathy and DR often co-occur as complications. Circadian rhythms influence the progression of both conditions through shared mechanisms, including inflammation, oxidative stress, and vascular dysfunction. In diabetic nephropathy, circadian disruptions worsen kidney damage by increasing fluctuations in GFR and enhancing oxidative stress (Olaoye et al., 2019; Firsov and Bonny, 2018b). Simultaneously, circadian misalignment negatively impacts retinal vascular health, accelerating DR progression (Liu WJ. et al., 2023). Key circadian genes regulate vascular factors like vascular endothelial growth factor (VEGF) and endothelial nitric oxide synthase (eNOS), which are crucial for endothelial function and vascular stability; disturbances in circadian rhythms impair endothelial function, increase vascular permeability, and lead to abnormal angiogenesis, collectively worsening both kidney and eye diseases (Bhatwadekar and Rameswara, 2020).

Modern lifestyle factors like shift work, irregular light exposure, and sleep disorders further underscore the co-morbid risk between kidney and eye diseases. Circadian misalignment from these lifestyle factors increases the risk of conditions such as glaucoma, DED, cataracts, and CDK (Huang et al., 2021; Uhm et al., 2018; Kara and Yilmaz, 2015). This misalignment promotes immune dysfunction, inflammation, and oxidative stress, contributing to kidney fibrosis and retinal degeneration (Huang et al., 2024a; Huang et al., 2023). Additionally, circadian disruption is associated with metabolic abnormalities that may drive the co-occurrence of diabetic nephropathy and DR. Growing evidence underscores the need for integrated therapeutic strategies aimed at restoring circadian alignment to improve kidney and eye health.

In summary, circadian rhythms are essential for regulating the physiological processes underlying both kidney and eye health. Shared mechanisms—such as inflammation, oxidative stress, fibrosis, and dysregulated pressure systems—connect these organs, increasing susceptibility to co-morbid conditions when circadian rhythms are disrupted. Recognizing these connections provides new insights into preventing and treating co-morbid kidney and eye diseases and highlights the potential benefits of therapeutic strategies focused on restoring circadian alignment.

## 6 Clinical implications and future directions

The growing understanding of the critical role that circadian rhythms play in regulating physiological processes in the kidney and eye opens new therapeutic avenues. Targeting circadian rhythms can enhance treatment efficacy and reduce side effects, potentially improving outcomes for patients with CKD, diabetic nephropathy, and ocular diseases. Promising strategies like chronotherapy, light therapy, time-restricted feeding, and pharmacological interventions are emerging to align treatments with circadian rhythms, potentially transforming kidney and eye health management.

### 6.1 Therapeutic approaches targeting circadian rhythms in kidney and eye diseases

Circadian rhythm disruption plays a critical role in the pathogenesis of kidney and ocular diseases by promoting inflammation, oxidative stress, fibrosis, and hormonal imbalance. Chronotherapy, which involves synchronizing therapeutic interventions with the body’s endogenous circadian clock, has emerged as a promising approach to mitigate these pathological processes.

In kidney diseases, chronotherapy is particularly relevant due to the circadian regulation of renal function and drug metabolism. For instance, pharmacologic agents such as angiotensin-converting enzyme (ACE) inhibitors and angiotensin receptor blockers (ARBs) exhibit time-of-day-dependent effects, optimizing blood pressure control and renal protection when administered in alignment with circadian fluctuations (Tsimakouridze et al., 2015; Solocinski and Gumz, 2015). Diuretics, another key therapeutic class, can reduce electrolyte imbalances and nocturia when administered at specific times that correspond with fluid regulation rhythms (Smolensky et al., 2021). Additionally, time-restricted feeding—a behavioral chronotherapy—has demonstrated renoprotective effects in experimental models by reducing systemic inflammation and metabolic dysregulation (Chaix et al., 2014; Hatori et al., 2012).

In ocular diseases, chronotherapy also holds substantial potential. In glaucoma, IOP follows a distinct diurnal pattern, and aligning the administration of IOP-lowering agents with peak IOP periods may enhance therapeutic efficacy and reduce optic nerve damage (Ciulla et al., 2020; Hermida and Ayala, 2016). Similarly, DR, AMD, and DED exhibit circadian fluctuations in ocular surface health, inflammatory responses, and tissue repair, suggesting potential windows of therapeutic intervention (Turner et al., 2010; Messmer, 2015). Light therapy, a non-pharmacological chronotherapeutic approach, has shown promise in resetting circadian rhythms and ameliorating retinal dysfunction in conditions like AMD and glaucoma (Zhu et al., 2021; Ahn et al., 2023).

Moreover, the timing of therapeutic interventions is not limited to pharmacological agents but also includes light exposure and dietary interventions. For instance, appropriately timed light therapy has been shown to re-entrain disrupted circadian rhythms and mitigate renal dysfunction in diabetic nephropathy (De Lavallaz and Musso, 2018; Thomas and Cooper, 2010). Similarly, in ocular diseases, light modulation can influence retinal circadian rhythms and reduce disease progression in AMD and glaucoma (Zhu et al., 2021; Ahn et al., 2023).

**FIGURE 5:**
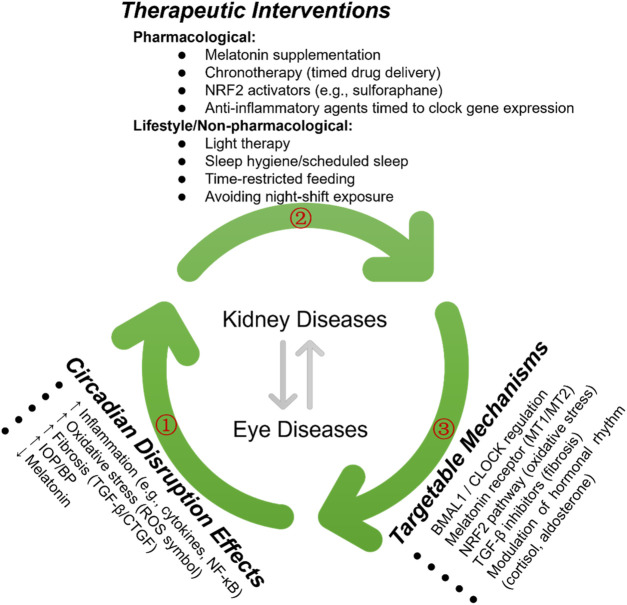
Therapeutic Strategies Targeting Circadian Rhythms in Kidney and Eye Diseases. The green arrows (①, ②, ③) illustrate the key regulatory pathways involved in circadian rhythm modulation and their therapeutic implications in kidney and eye diseases. The bidirectional arrows represent reciprocal interactions between the pathways, highlighting potential intervention targets.

To further elucidate the role of chronotherapy in kidney and ocular diseases, we have included a new schematicthat integrates the pathological effects of circadian disruption, corresponding molecular targets, and therapeutic strategies under investigation (Figure 5). This visual representation underscores the importance of chronotherapy as a multifaceted approach encompassing pharmacological, behavioral, and light-based interventions to restore circadian alignment and improve clinical outcomes.

### 6.2 Pharmacological interventions

Emerging pharmacological strategies targeting molecular components of the circadian system are gaining momentum, with recent advances in both preclinical models and early clinical trials. Key therapeutic targets include BMAL1/CLOCK transcriptional regulation, melatonin receptor signaling (MT1/MT2), oxidative stress pathways (e.g., NRF2 activation), and fibrotic cascades (e.g., TGF-β inhibition). Notably, several classes of small-molecule modulators have demonstrated translational potential in circadian-related pathologies.

#### 6.2.1 REV-ERB agonists and antagonist

Synthetic agonists such as SR9009 and SR9011 act through nuclear receptors REV-ERBα/β to repress BMAL1 expression and modulate circadian amplitude. Preclinical studies reveal their dual therapeutic effects: in metabolic disease models, SR9009 reduces systemic inflammation and improves glucose homeostasis (Hong et al., 2021; Wang et al., 2020), while in renal fibrosis models, it attenuates TGF-β-mediated collagen deposition (Takaguri et al., 2025). A synthetic small molecule compound antagonist SR8278 targeting Rev-erb-α/β can limit ferroptosition in the AKI model induced by folic acid or aristolochic acid and alleviate the injury (Guo et al., 2021; Wang et al., 2021). Ocular studies further suggest REV-ERBα agonism protects retinal neurons from oxidative stress, potentially delaying photoreceptor degeneration in retinopathies (Huang et al., 2022b). Our recent work extends these findings, demonstrating that REV-ERB modulation via SR8278 rescues light-cycle disruption-induced lacrimal gland dysfunction, highlighting its ocular surface protective effects (Huang et al., 2024b).

#### 6.2.2 ROR pathway modulators

The RORγ inverse agonist SR1078 exemplifies circadian-targeted immunomodulation. By suppressing ROR-driven BMAL1 transcription, it reduces IL-17 production in autoimmune models–a mechanism particularly relevant for uveitis and lupus nephritis (Wang et al., 2010; Ribeiro et al., 2021). Emerging evidence suggests ROR modulators may concurrently address oxidative damage through NRF2 pathway crosstalk (Cabrera-Serrano et al., 2025), though clinical validation remains pending.

#### 6.2.3 Melatonin receptor agonists

Clinically approved agents like ramelteon and agomelatine bridge circadian pharmacology with therapeutic practice. Beyond their established use in sleep disorders, ramelteon alleviates experimental acute ocular inflammation via HIF-1Α/VEGF/E-NOS signaling (Usta Sofu et al., 2022), while agomelatine attenuates cisplatin-induced nephrotoxicity by suppressing NF-κB-driven inflammation (Dil et al., 2023).

These advances are supported by systematic pharmacological reviews from our team and others (Ruan et al., 2021; Huang et al., 2020). While most compounds remain in preclinical development, first-in-human trials of Melatonin (NCT03478306, NCT05701969, and NCT02642640) signal growing clinical translation. Critical challenges include tissue-specific delivery optimization and circadian-timed dosing regimens to maximize therapeutic efficacy while minimizing off-target effects.

### 6.3 Translational potential

Significant progress in understanding circadian regulation of kidney and eye health has been made, yet considerable gaps remain in translating these findings into clinical practice. A primary challenge is the lack of integrated models that assess circadian disruption in both kidney and eye simultaneously. Current research often focuses on each organ separately, limiting insights into how circadian misalignment in one tissue may affect the other. A comprehensive approach is needed to explore systemic effects of circadian disruption and organ-specific therapies targeting shared molecular pathways in kidney and ocular diseases.

Cross-disciplinary collaboration among chronobiology, nephrology, ophthalmology, and bioinformatics is essential to accelerate identifying novel therapeutic targets and biomarkers. For instance, PER and BMAL1, key regulators of circadian rhythms in both kidney and eye, could serve as biomarkers for disease monitoring and treatment efficacy (Firsov and Bonny, 2018b; Zhang and Jie, 2024). Examining how these biomarkers fluctuate in response to therapies could offer valuable insights for treatment optimization. Moreover, advancements in genomic and proteomic technologies may reveal patient-specific circadian patterns, facilitating precision medicine tailored to individual circadian profiles. For instance, in patients with diabetic nephropathy and DR, personalized therapies aligned with their circadian rhythms could improve efficacy and reduce side effects (McMahon et al., 2014; Bhatwadekar and Rameswara, 2020; Wu et al., 2023).

Integrating biomarkers and precision medicine into treatment regimens will be crucial to advance clinical applications of circadian-based therapies. Biomarkers reflecting circadian gene expression or timing disruptions could identify at-risk patients, monitor disease progression, and guide intervention timing. Ultimately, these biomarkers could be integrated into routine clinical care, offering more accurate and effective treatments for kidney and eye disease patients (Tummalapalli et al., 2016; Suárez-Cortés et al., 2022).

Circadian-targeted therapies represent a novel, promising strategy for managing kidney and ocular diseases. Circadian-based therapies—including chronotherapy, light therapy, and time-restricted feeding—offer promising strategies for managing kidney and ocular diseases. By aligning treatment regimens with endogenous biological rhythms, these interventions may enhance therapeutic efficacy, minimize adverse effects, and allow for more personalized treatment approaches, particularly in patients with comorbid conditions like CKD and eye disorders such as glaucoma, DR, and DED. However, additional research is needed to translate these approaches into clinical practice. Bridging research gaps, developing integrated models for circadian disruption assessment, and incorporating precision medicine and biomarkers into treatment regimens will be crucial to fully realize circadian-based therapies' potential.

## 7 Conclusion

Circadian rhythms orchestrate critical physiological processes that maintain kidney and eye health, including blood pressure regulation, IOP balance, cellular repair, and fluid homeostasis. Disruptions to these rhythms—caused by factors such as shift work, irregular sleep, or artificial light exposure—contribute to the onset and progression of CDK, diabetic nephropathy, glaucoma, DED, and DR.

This review highlights the shared pathological pathways—oxidative stress, inflammation, and fibrosis—linking renal and ocular conditions, emphasizing the importance of circadian synchronization as a unifying therapeutic target. Integrating circadian biology into disease management could enhance both preventive and therapeutic strategies, particularly for patients with co-morbid kidney and eye disorders.

Emerging circadian-aligned interventions, such as chronotherapy, light therapy, and time-restricted feeding, offer promising, personalized treatment options by restoring rhythmic homeostasis. Moving forward, multidisciplinary research is needed to develop robust biomarkers of circadian disruption and to validate rhythm-targeted therapies across organ systems.

By bridging insights from nephrology and ophthalmology, this review advocates for a holistic, circadian-centered approach to managing complex systemic diseases.

## Abbreviations

CKD, Chronic Kidney Disease; SCN, Suprachiasmatic Nucleus; TTFLs, Transcription-Translation Feedback Loops; IOP, Intraocular Pressure; AMD, Age-related Macular Degeneration; AKI, Acute Kidney Injury; DED, Dry Eye Disease; RAAS, Renin-Angiotensin-Aldosterone System; ROS, Reactive Oxygen Species; SNS, Sympathetic Nervous System; VIP, Vasoactive Intestinal Peptide; VEGF, Vascular Endothelial Growth Factor; eNOS, Endothelial Nitric Oxide Synthase; DR, Diabetic Retinopathy; TGF-β, Transforming Growth Factor-Beta; CTGF, Connective Tissue Growth Factor; BMAL1, Brain and Muscle ARNT-Like 1; CRY, Cryptochrome; PER, Period; CLOCK, Circadian Locomotor Output Cycles Kaput; ACE, Angiotensin-Converting Enzyme; ARBs, Angiotensin Receptor Blockers; RHT, Retinohypothalamic Tract; RPE, Retinal Pigment Epithelium; HIF, Hypoxia-Inducible Factor; GFR, Glomerular Filtration Rate; NF-κB, Nuclear Factor Kappa-light-chain-enhancer of Activated B cells.
